# Characterization of a Familial Goldenhar Syndrome Case Using Whole-Exome Sequencing

**DOI:** 10.3390/genes17030299

**Published:** 2026-02-28

**Authors:** Yosra Bejaoui, Yasser Al-Sarraj, Jana Al-Hage, Fadi F. Bitar, Nady El Hajj, Georges Nemer, Mazen Kurban

**Affiliations:** 1 College of Health and Life Sciences, Hamad Bin Khalifa University, Qatar Foundation, Doha P.O. Box 34110, Qatar; yob4003@qatar-med.cornell.edu (Y.B.);; 2 Weill Cornell Medicine, Doha P.O. Box 24144, Qatar; 3 Qatar Genome Program (QGP), Qatar Foundation Research, Development and Innovation, Qatar Foundation (QF), Doha P.O. Box 5825, Qatar; yalsarraj@qf.org.qa; 4 Department of Dermatology, American University of Beirut Medical Center, Beirut P.O. Box 11-0236, Lebanon; ja90@aub.edu.lb; 5 Department of Pediatrics and Adolescent Medicine, American University of Beirut Medical Center, Beirut P.O. Box 11-0236, Lebanon; fadi.bitar@aub.edu.lb; 6 College of Science and Engineering, Hamad Bin Khalifa University, Qatar Foundation, Doha P.O. Box 34110, Qatar; 7 Department of Biochemistry and Molecular Genetics, American University of Beirut, Beirut P.O. Box 11-0236, Lebanon

**Keywords:** genetic variant, clinical observation, diagnosis, Goldenhar disease

## Abstract

Background: Goldenhar syndrome (oculo–auriculo–vertebral spectrum, OAVS) is a rare congenital disorder characterized by craniofacial malformations, systemic anomalies, and significant phenotypic variability. Although it is the second most common craniofacial malformation after a cleft palate, the genetic etiology of Goldenhar syndrome remains largely unexplored. This study aimed to identify genetic variants contributing to Goldenhar syndrome in a Lebanese family with three affected individuals, using whole-exome sequencing and complementary genomic approaches. Methods: Whole-exome sequencing was performed on the nuclear family to identify variants associated with the syndrome. Complementary DNA methylation and gene ontology analyses were conducted to explore epigenetic modifications. Results: A missense shared variant in the *MID1* between the affected individuals [NP_000372.1): p. Ile593Phe] gene was observed in the family, while current ACMG evidence was insufficient to establish causality. Additional variants were identified, including a de novo mutation in *FBXW11* and a rare frameshift alteration in *NDUFAF8*, with limited segregation, implicating these genes in associated phenotypes such as craniofacial anomalies and cardiac defects. DNA methylation analysis revealed hypomethylation at CpG sites within the *ZC3H3* gene, suggesting an epigenetic contribution to disease variability. Conclusions: Our findings underscore the genetic and epigenetic complexity of Goldenhar syndrome, providing new insights into its molecular etiology and highlighting the challenges of variant interpretation in familial cases of rare congenital disorders.

## 1. Introduction

Goldenhar syndrome [OMIM 164,210] is a rare congenital developmental disorder involving the first and second branchial arches derivatives, affecting mainly the ears, eyes, mandible, and vertebrae, as first described by Dr. Maurice Goldenhar in 1952 [1]. It is a non-random association of heterogeneous clinical phenotypes, such as microtia, hemifacial microsomia with mandibular hypoplasia, ocular epibulbar dermoid, and cervical vertebral malformations, which extend to include cardiac, renal, and central nervous system malformations [1,2,3]. In 1963, Gorlin suggested the name oculo–auriculo–vertebral (OAVS) dysplasia for this condition. The frequency of congenital heart disease in OAVS ranges from 5% to 58%, with conotruncal and septal defects being the most reported malformations [4]. Its incidence has been estimated at 3.8 per 100,000 births based on the European registry [5]. It is the second most frequent craniofacial malformation disorder after a cleft palate and one of the latest recurrent polymalformative syndromes [6]. It is a complex disease with various environmental and genetic etiologies described. Most cases of Goldenhar syndrome are sporadic; however, in rare cases, inheritance has been reported in an autosomal dominant pattern. Abnormalities of chromosomes and neural crest cells, as well as environmental factors during pregnancy, like ingestion of drugs and intake of alcohol by the mother, were also related to the development of the disease [7]. Twin pregnancies and artificial reproductive techniques have been considered as possible risk factors for OAVS [8,9]. Identifying new genes associated with Goldenhar syndrome is a crucial step in understanding the underlying pathophysiology of this complex disorder. To uncover the genetic basis of this disease, we investigate an extended family with six individuals presenting varying clinical features of the Goldenhar phenotype by performing whole-exome sequencing (WES) and DNA methylation profiling on a nuclear family, including three affected children and their parents.

## 2. Materials and Methods

### Research Participants and Clinical Data

**Figure 1:**
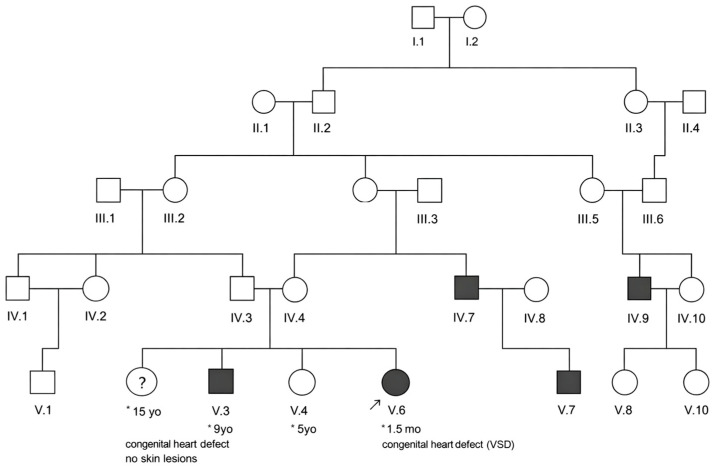
Family pedigree of patient cohort showing the family pedigree of six members with Goldenhar syndrome. I, II, III, IV, and V represent generations. The numbers below the boxes are the age at diagnosis. Double lines indicate consanguineous unions. The DNA samples were collected only from IV.5, IV.6, V.3, V.4, and V.6. * Age.

A three-generational family with multiple members experiencing Goldenhar syndrome was identified at the American University of Beirut Medical Center (AUBMC) Figure 1. The Institutional Review Board (IRB) at AUBMC reviewed and approved the study (Protocol Number: DER.MK.01). Written informed consent for genetic studies was obtained before collecting blood for DNA extraction.

DNA was collected from the nuclear family of three affected children (IV.5, IV.6, V.3, V.4, V.6). The parents (IV.5 and IV.6) are first cousins and reported no hearing loss or craniofacial abnormalities (Figure 1). The V.3 member is a 9-year-old female with accessory tragi bilateral, preauricular pits, hearing loss, and ocular dermoid. Her younger brother, V.4, is 6 years old with accessory tragi and preauricular pits, and the proband younger sister, V.6, is a 6-week-old girl with multiple accessory tragi bilateral, microtia, preauricular pits, and a moderate ventricular septal defect. Additional affected individuals (IV.9, IV.7, V.7) were identified within the extended pedigree; however, biological samples were unavailable for genetic analyses, and their inclusion has been based on family reports. Notably, consanguineous unions were present in (IV.3–IV.4 and IV.9–IV.10), indicating that consanguinity is recurrent within this family.

**Figure 2:**
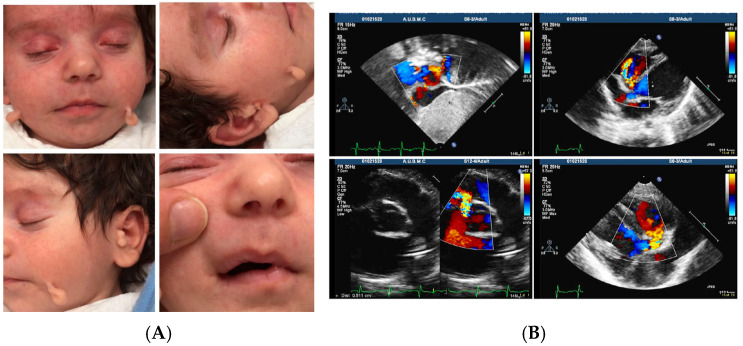
(**A**): Multiple bilateral accessory tragi and right-sided perioral pits in a 6-month-old girl with Goldenhar syndrome. (**B**): Echocardiogram with color Doppler imaging of V.6 showing a moderately sized premembranous ventricular septal defect, trace aortic and mitral insufficiencies, and physiological tricuspid and pulmonary insufficiency.

The V.6 family member underwent comprehensive phenotyping and diagnosis, showing multiple accessory tragi in front of the ears bilaterally and along the jawline, as seen in Figure 2A. At the time of the examination, V.6 was 6 weeks old with a blood pressure of 93/47 mmHg, height of 52 cm, and weight of 4.2 kg. The AUBMC Children’s Heart Unit echocardiography showed levocardia and atrial situs solitus. The relationship of the cardiac chambers to each other and to the great vessels was normal. The left ventricle (LV) was mildly dilated with a minimal decrease in LV systolic function. The other cardiac chambers were standard in size, thickness, and systolic function. Some pulmonary venous return to the left atrium was seen. A moderate-sized perimembranous ventricular septal defect was detected, measuring 5 × 4.5 mm in dimensions with aneurysmal tissue formation. There was a tiny patent foramen ovale. The cardiac valves appeared normal. The outflow tracts were patent. The pulmonary artery and its branches were of normal size. The aortic arch was left-sided without coarctation. The color Doppler study demonstrated trace aortic and mitral insufficiencies, as well as physiological tricuspid and pulmonary insufficiency, as seen in Figure 2B. All patients underwent a comprehensive otolaryngologic examination and pure tone audiometry testing in soundproof rooms at the AUB Medical Center (AUBMC). They were also referred to ophthalmology, cardiology, and nephrology to identify other possible congenital abnormalities and to rule out syndromic hearing loss.

Exome Sequencing:

DNA samples (IV.5, IV.6, V.3, V.4, V.6) were shipped to Macrogen (Seoul, Republic of Korea), where library preparation, exome capture, sequencing, and data analysis were performed using Sureselect V6-Post Enrichment workflow, with sequencing on Illumina HiSeq2000/2500 (Illumina, San Diego, CA, USA). VCF files were generated using the GATK workflow; their quality assessment is present in Supplementary Table S1. VCF analysis was conducted using QIAGEN Clinical Insight (QCI 9.3.2).) Interpret (QIAGEN, Germantown, MD, USA), applying a preconfigured workflow with the WGS/WES Rare Disease-Standard Filtering protocol (QIAGEN). QCI Interpret classified variants by pathogenicity (e.g., pathogenic, likely pathogenic, benign, or of uncertain significance) supported by the structured content from the QIAGEN Knowledge Base. QCI Interpret evaluated variants by automatically triggering evidence across the ACMG/AMP criteria, assigning weighted evidence strengths and integrating case-level information, including inheritance models and variant segregation across analyzed samples, based on the curated literature and database-supported evidence from the QIAGEN Knowledge Base [10].

In the workflow applied to our cohort, variants with an allele frequency greater than or equal to 1.0% were excluded unless they were established as pathogenic. Then, the exonic and gene-associated structural variants experimentally linked to a phenotype were retained. These included variants classified as pathogenic, possibly pathogenic, or disease-associated according to the Human Gene Mutation database (HGMD)or those documented in the literature as gain-of-function mutations; gene fusions/rearrangements; overlapping with copy number gains; frameshifts, in-frame indels; stop codon changes; missense variants; splice site variants (within two bases into the intron); or those predicted to disrupt splicing by MaxEntScan. Variants overlapping with copy number losses were also retained [11,12].

SNP genotyping:

Deep SNP genotyping was conducted on Infinium Omni 2.5 with the family members [III.1, IV.4, IV.2, IV.5, IV.6, V.3, V.4, V.6], and analysis was performed using Genome Studio v2.0.5.

Primary quality control was performed using the Reproducibility and Heritability Report from GenomeStudio. CNVPartition (Illumina, GenomeStudio, 2021) methods were subsequently used to discover the CNVs in the datasets. The methods aimed to detect CNVs based on the Log R Ratio (LRR) deviation status and the B Allele Frequency (BAF). The analysis was performed using the default configurations (Illumina, GenomeStudio, 2021).

DNA methylation profiling:

DNA methylation profiling was performed using the Illumina Infinium MethylationEPIC v1.0 BeadChip. Genomic DNA (~500 ng) from three affected family members (V.3, V.4, and V.6) and three age-matched controls were used, of which one related control (V.1) and two unrelated individuals with no known congenital anomalies were bisulfite converted using the EZ DNA Methylation Kit (Zymo Research, Irvine, CA, USA). Converted DNA was then amplified, enzymatically fragmented, and hybridized to the arrays following the manufacturer’s instructions. The arrays were scanned using the Illumina iScan system, and raw intensity data (IDAT files) were processed in R using the RnBeads package [13]. Primary quality control and preprocessing steps were conducted, which excluded probes overlapping SNPs (*n* = 17,371) and probes with the highest fraction of unreliable measurements using greedycut (*n* = 1600); in total, 18,971 probes were removed, and all samples were retained, then data normalizing was performed using Dasen which was followed by excluding probes located on sex chromosomes (*n* = 19,033). Overall, 825,906 probes were retained for further differential DNA methylation analysis using the limma package, adjusting for age and sex [14,15]. Significant CpGs were then tested for gene ontology (GO) enrichment using the Gometh R package (R version 4.5.2) to perform gene set analysis following an adjustment for the number of CpG sites per gene [16].

## 3. Results

**Figure 3:**
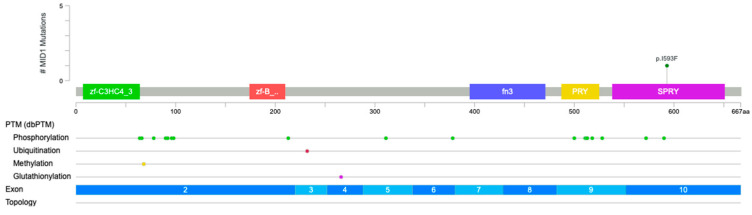
*MID1* mutation, p.I593F, is a missense alteration within the critical and well-established SPRY functional domain.

**Figure 4:**
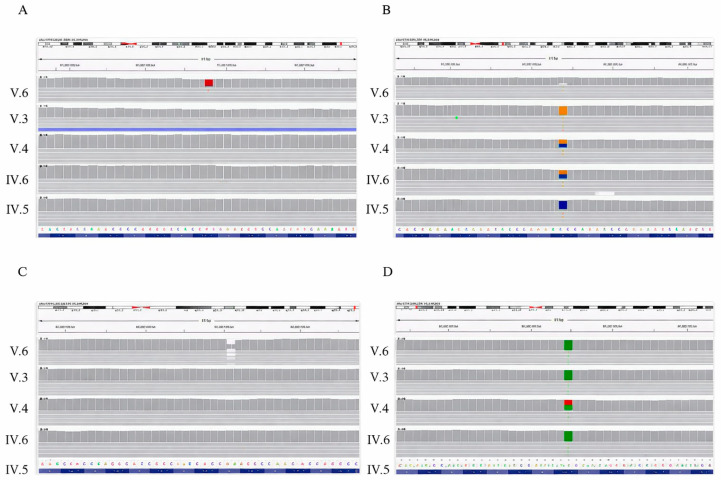
Integrative genomics viewer (IGV) images of specific mutations identified: (**A**) de novo heterozygous variant *FBXW11* [c.404T>A, p.M135K] in individual V.6; (**B**) homozygous missense alteration in *KMT2D* [c.8285G>C, p.G2762A] in individual V.3; (**C**) frameshift alteration in *NDUFAF8* [c.139del, p.S47Vfs*] in individual V.6; and (**D**) the *MID1* missense mutation, p.I593F.

We performed whole-exome sequencing (WES) for the three affected children [V.3, V.4, V.6] and their parents [IV.5, IV.6]. VCF files were analyzed using QCI Qiagen (QCI- 9.3.2). Starting with 42,175 variants across 14,114 genes, we excluded those with an allele frequency greater than or equal to 1.0% unless they were established as pathogenic. After this initial filtration, 2930 variants remained. We then applied an additional filter that resulted in 1477 variants, of which seven were classified as pathogenic but did not conform to the autosomal recessive or dominant inheritance model and were therefore excluded. We then restricted the analysis to variants that were either homozygous or hemizygous and present in at least three affected individuals at the gene level. Following this step, nine variants remained: five classified as benign, one likely benign, four with conflicting interpretations, and three of uncertain significance (VUS) according to QCI Qiagen (Table 1). Among the variants of uncertain significance, a missense *MID1* variant NM_000381.4 (NP_000372.1): p. Ile593Phe, resulting in a substitution from isoleucine to phenylalanine within the critical and well-established SPRY functional domain, was identified (Figure 3 and Figure 4). The variant is absent from the gnomAD database; however, according to ACMG/AMP criteria and available in silico predictions, current evidence is insufficient to support pathogenicity. Although computational scores such as CADD can suggest a potential functional impact, no experimental or segregation data from extended family member were available to establish a causal role.

MID1 variations are associated with Opitz G/BBB syndrome; while this syndrome has distinct genetic causes compared to Goldenhar syndrome, they can present with overlapping symptoms, particularly craniofacial anomalies and midline defects. In the presented patients, the overlapping features included ear abnormalities, such as microtia or accessory tragi, and congenital heart defects (CHDs), such as septal or outflow tract anomalies. This variant was heterozygous in the mother and hemizygous in the father (Figure 4); despite the observed phenotypic overlap, the MID1 variant was interpreted as a variant of uncertain significance, and its potential contribution to the observed phenotypes remains speculative pending further genetic and functional studies.

Following the identification of a shared variant among the three affected individuals, we investigated additional variants that may correspond to specific clinical phenotypes, such as hearing loss, and congenital heart defects, such as septal defects, present in V.3 and V.6.

In the individual V.6, a de novo variant heterozygous FBXW11[NM_001378974.1(NP_001365903.1): p.(Met135Lys)] was identified which was absent from the Genome Aggregation Database (gnomAD); population databases have reportedly suggested the biological relevance of de novo variants in this gene, as they were shown to be associated with neurodevelopmental, jaw, eye, and digital syndromes [17]. In addition, we identified a frameshift alteration in *NDUFAF8* [NM_001086521.2:c.139del:p. Ser47ValfsTer58] where nonsense-mediated decay (NMD) was predicted, and the exon was present in a biologically relevant (MANE) transcript. In individual V.3, we observed a homozygous missense variant in *KMT2D* (NM_003482.4(NP_003473.3):p.(Gly2762Ala). In silico scores suggested a possible functional impact with a CADD score of 22.7 with a PolyPhen classification as “probably damaging”. The variant is rare and absent from gnomAD. These variants were unique to this family, and were not found in gnomAD or any patient from Lebanon in more than 300 exomes (Table 2).

It is essential to highlight that we also investigated variations in genes *MYT1*, *AMIGO2*, *ZYG11B*, *SF3B2*, *EYA3*, *VWA1*, *ZIC3*, *OTX2*, *YPEL1,* and *PTCH2*, which were previously proposed to be associated with Goldenhar syndrome in other studies [18,19,20,21,22,23,24,25,26]. Among these, we identified only a heterozygous in-frame deletion in *MYT1* (NM_004535.3(NP_004526.1): p.Glu306del) located in a repetitive region without a well-defined function. This variant has a relatively high allele frequency of 3.2674% in the gnomAD Ashkenazi Jewish population, and it was present in the affected individuals V.3 and V.6 as well as the unaffected mother IV.6, indicating that it had unlikely contributed to the phenotype in this family.

**Figure 5:**
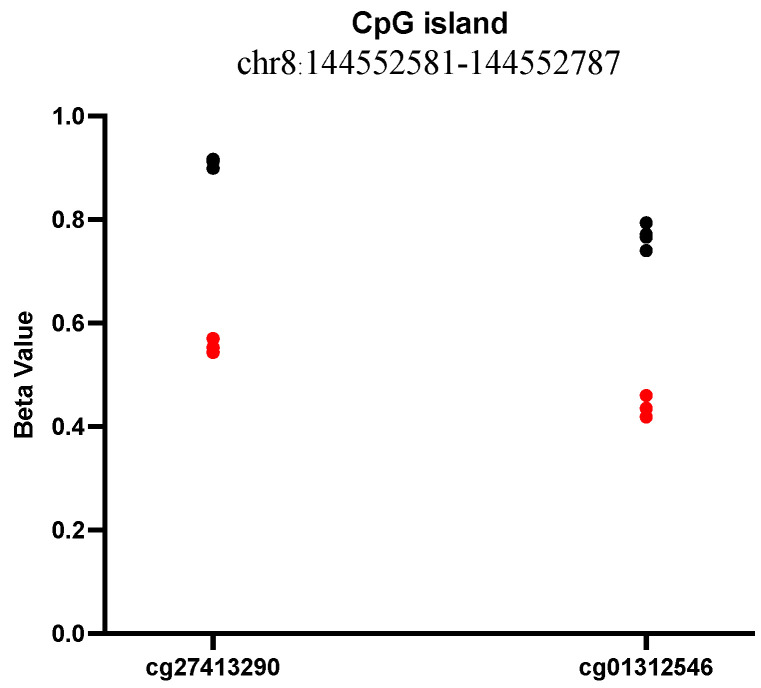
Methylation findings in Goldenhar family. A significant CpG island harboring two CpG sites in the *ZC3H3* gene was hypomethylated in Goldenhar compared to the control. Red dots represent the affected children; black dots represent the controls.

We then investigated copy number variations using microarray-based analysis in Infinium Omni 2.5; we did not reveal any pathogenic or clinically relevant CNVs in the affected individuals. DNA methylation profiling was subsequently performed using the Illumina EPIC array V1; 45 CpG sites, eight tiling, and one CpG island were found to be significant (Supplementary Table S2). We then performed gene ontology (GO) enrichment analysis for the 45 CpG sites; terms such as junctional sarcoplasmic reticulum membrane, regulation of ryanodine-sensitive calcium-release channel activity, glutaminyl–peptide acyltransferase activity, and cytoskeletal regulatory protein-binding were significant at a nominal *p*-value (Supplementary Table S3). The one significant CpG island contained two CpG sites spanning the *ZC3H3* gene, which were hypomethylated in Goldenhar compared to controls (Figure 5). Although these pathways did not directly implicate previously established Goldenhar syndrome genes, they may reflect broader disruptions in developmental or cellular signaling processes.

## 4. Discussion

Here, we described three affected children from an extended family exhibiting variation in their clinical manifestation of Goldenhar syndrome. We utilized genomic analysis to uncover the disorder’s genetic basis and the observed diversity of clinical manifestations. Among the identified variants, a shared missense variant within the affected individuals in the *MID1* gene, p.I593F, within the critical and well-established SPRY functional domain was identified. Mutations in the MID1 gene have been associated with the Opitz G/BBB syndrome, characterized by multiple congenital midline structure anomalies such as hypertelorism, frontal bossing, a broad nasal bridge, and a cleft lip. This syndrome has overlap with Goldenhar syndrome, mainly in the craniofacial anomalies, which raises the possibility that MID1 dysfunction may contribute to atypical or variant forms of Goldenhar syndrome. This variability could be due to the tissue-specific roles of MID1, as the MID1 mutation affects neural crest cells which give rise to almost all cell types of ectodermal and mesodermal origin [27], and/or to the mild effect the missense variant has on the protein function and structure as per the in silico predictive tool outcomes (Supplementary Table S4).

Although partial overlap exists with Goldenhar syndrome, current genetic and segregation data in this family were insufficient to support a causal role for the identified *MID1* variant. Previous studies have reported variable expressivity of MID1 where it was shown that MID1 mutations can demonstrate low penetrance in males; as described by Ruiter et al., a boy with a relatively mild form of Opitz G/BBB syndrome carried p.Lys370Glu (c.1108A>G) the mutation in *MID1*, and this mutation was found in his clinically affected brother as well as in the healthy maternal uncle [28]. While such observations may provide a possible context for phenotypic variability in male carriers within the present pedigree, definitive interpretation requires genetic evidence such as extended segregation analysis or linkage studies using X-chromosome markers.

In addition to the shared *MID1* variant, additional rare variants were identified. A de novo *FBXW11* variant, previously implicated in craniofacial and ocular development, was identified in the individual V.6 [17]. Similarly, a frameshift variant in *NDUFAF8*, a gene involved in mitochondrial complex I assembly, may explain the cardiac abnormalities observed in the affected member V.6, as it was shown to be associated with diseases such as Leigh-like Encephalomyopathy that cause cardiac hypertrophy [29]. A missense variant in *KMT2D*, a gene associated with Kabuki syndrome, may account for some of the auditory and developmental features reported in the individual V.3 [30].

DNA methylation profiling identified 45 CpG sites, eight tiling, and one CpG island that were significant. The CpG islands harbored two hypomethylated CpG sites in the effected individuals, which spans the ZC3H3 gene and encodes a zinc-finger protein with potential roles in transcriptional regulation [31]. Pathway analysis revealed several biologically relevant pathways at a nominal *p*-value associated with the junctional sarcoplasmic reticulum membrane, ryanodine-sensitive calcium-release channel activity regulation, and cytoskeletal regulatory protein binding. These identified pathways could provide additional insight into the molecular mechanisms underlying the development of craniofacial and cardiac anomalies seen in Goldenhar syndrome. For example, the dysregulation of calcium signaling via the sarcoplasmic reticulum has been shown to affect muscle and tissue development, contributing to congenital heart defects and other musculoskeletal anomalies observed in our patients [32]. The absence of relevant copy number variations (CNVs) detected by microarray analysis further supported the hypothesis that these single-nucleotide variants were likely the primary genetic contributors to the syndrome in this family. These findings highlight the multifactorial nature of the phenotype observed in this family and have shown the importance of considering genetic and epigenetic factors in the interpretation of complex congenital syndromes.

## 5. Conclusions

In conclusion, our comprehensive genomic and epigenomic analyses identified several multiple variants in genes, such as *MID1*, *FBXW11*, *NDUFAF8*, and *KMT2D*, that may contribute to the phenotypic presentation of Goldenhar syndrome in this family. However, the available genetic evidence and ACMG/AMP-based interpretations do not support a definitive pathogenic role for most of the variants in this family. The interpretation of variants was limited by incomplete segregation analysis due to the unavailability of DNA samples from extended family members, and further functional studies are necessary to confirm the pathogenicity of these variants. Our findings broaden the genetic heterogeneity associated with Goldenhar syndrome and highlight the importance of considering diverse genetic factors in its etiology.

## Figures and Tables

**Table 1:** Variants of uncertain significance after genetic filtration using QIAGEN^®^ Clinical Insight Interpret.

Gene	Alteration	HGVS Nomenclature	Phenotype	Mode of Inheritance	Impact	CADD Score	Max Population Frequency	ACMG/AMP
*SPDL1*	c.878T>C	NM_017785.5(NP_060255.3): p.(Met293Thr)	Hereditary Disorder	-	Missense	25.9	0.0764% gnomAD (Latino)	VUS (BP1, PM2, BP4)
	p.M293T							
*MID1*	c.1777A>T	NM_000381.4 (NP_000372.1): p. Ile593Phe	Opitz G/BBB syndrome	X-linked	Missense	17.75	0% gnomAD	VUS (PM1, PM2, BP4)
	p.I593F							
*ARSH*	c.109C>T	NM_001011719.2(NP_001011719.1): p.(Arg37Cys)	Cancers and Tumors	-	Missense	18.77	2.4773% gnomAD (South Asian)	Benign (BS1, BS2)
	p.R37C							

**Table 2:** Rare variants identified in affected individuals after genetic filtration and ACMG/AMP classification.

Individual	Gene	HGVS	Variant Type	Associated Phenotype	Population Frequency	ACMG/AMP Classification
V.6	*FBXW11*	NM_001378974.1:c.404T>A/p.M135K	Missense	Neurodevelopmental disorder with craniofacial, ocular, and limb anomalies	Absent gnomAD	Likely pathogenic (PM1, PM2, PP2, PP3)
V.6	*NDUFAF8*	NM_001086521.2:c.139del/p.S47Vfs*58	Frameshift	Mitochondrial complex I deficiency	Absent gnomAD	Likely pathogenic (PVS1, PM2)
V.3	*KMT2D*	NM_003482.4:c.8285G>C/p.G2762A	Missense	Kabuki syndrome	Absent gnomAD	VUS (PM2, PP2)

## Data Availability

Exomes sequencing raw data (Fastq files) were uploaded to the National Center for Biotechnology Information (NCBI) portal and registered under the reference number BioProject PRJNA1198722).

## Supplementary Materials

The following supporting information can be downloaded at: https://www.mdpi.com/article/10.3390/genes17030299/s1, Table S1: Quality control assessment for whole exome sequencing data, Table S2: Significant Methylation Alterations in Goldenhar Patients, Table S3: Gene Ontology Analysis of Goldenhar Family Members Methylation Results, Table S4: In Silico predictions for the MID1 missense variant.
